# Stillbirths and hospital early neonatal deaths at Queen Elizabeth Central Hospital, Blantyre-Malawi

**DOI:** 10.1186/1755-7682-2-25

**Published:** 2009-08-31

**Authors:** Aklilu M Metaferia, Adamson S Muula

**Affiliations:** 1 Formerly, Department of Obstetrics and Gynecology, College of Medicine, Blantyre, Malawi; 2 Department of Public Health, Division of Community Health, College of Medicine, Blantyre, Malawi

## Abstract

**Background:**

Much of the data on still births and early neonatal deaths from resource-limited settings are obtained via maternal recall from national or community level surveys. While this approach results in useful information to be obtained, often such data suffer from significant recall bias and misclassification. In order to determine the prevalence of stillbirths (SB), early hospital neonatal death (EHND) and associated factors in Blantyre, Malawi, a prospective study of pregnant and post-natal women was conducted at the Queen Elizabeth Central Hospital (QECH), Malawi.

**Methods:**

A prospective observational study was conducted between February 1, 2004 and October 30, 2005. Consecutive women attending the hospital for delivery were recruited. Data were collected on the health status of the fetus on admission to labor ward and immediately after delivery, whether alive or dead. Gestational age (GA) and birth weight (BW) and sex of the newborn were also noted. Similar data were also collected on the live births that died in the delivery room or nursery. Data were analyzed using SPSS (Statistical Package for the Social Sciences) statistical package.

**Results:**

A total of 10,700 deliveries were conducted during the 12 months study period and of these deliveries, 845 (7.9%) were SB and EHND. Stillbirths comprised 3.4% of all deliveries; 20.2% of the ante-partum deaths occurred before the mother was admitted to the labor ward while a slightly higher proportion (22.7%) of fetal loss occurred during the process of labor and delivery. Fifty-sex percent of the perinatal deaths (PD) were EHND. The mean gestational age for the perinatal deaths was 34.7 weeks and mean birth weight was 2,155 g (standard deviation = 938 g). The majority, 468 (57.8%) of the perinatal deaths were males and 350 (43.2%) were females. Many of the perinatal deaths (57.9%) were deliveries between gestational ages of 20 and 37 weeks. Most (62.7%) of the mothers with a perinatal death had experienced a previous similar incident.

**Conclusion:**

About 3.4% of all pregnant mothers past 20 weeks of gestation ended up in delivering a stillbirth; another 4.4% of the live births died before discharge from hospital, thus, 7.9% of pregnancy loss after 20 weeks (or 500 g estimated weight) of gestation. This is a higher loss when compared to international and regional data. We recommend attention be given to these unfavorable outcomes and preventive measures or intervention for preventable causes be considered seriously. These measures could include the provision of emergency obstetric care, improving access to deliveries by health professionals and resourcing of health facilities such that neonatal viability is promoted.

## Background

The rates of stillbirths (SB) and early neonatal deaths (ENND) may be considered as indicators of the quality of ante-partum, intra-partum and neonatal care [1,2]. Garne [3] has however argued that perinatal deaths are not a good indicator of medical care. Data on the frequency and distribution of adverse birth outcomes are important for planning and execution of maternal and child health care intervention services in developing countries; information on local patterns of SB and ENND will be helpful in improving perinatal care at the local level.

The estimated incidences of SB and ENND vary worldwide based on socio-economic, demographic and clinical profiles. An earlier report from Malawi reported a perinatal death rate of 68.3 per 1000 births; 56% were SB and the remaining 44%, early neonatal deaths [4]. In Kenya, among studied perinatal deaths, antepartum deaths comprised 23%, intrapartum deaths (fresh stillbirth) 38%, and neonatal deaths within 24 hours following birth at 39% [5]. Investigators from Nigeria reported a perinatal mortality rate of 77.03 per 1000 total births; of these 73.5% were SB and the remaining 26.5%, early neonatal deaths [6].

In low income countries a significant proportion of SB occurs in the intrapartum period and these deaths are commonly attributed to avoidable factors related to inadequate maternity care. In contrast, in developed nations these deaths are largely pre-partal with no apparent cause [7]. Deprived societies in developed nations however, may suffer similar impediments to maternity care and adverse outcomes as are observed in resource-limited nations [8-10].

We undertook this prospective observational study to estimate the incidence of SB and END as well as assess how these relate to gestational age, birth weight and sex of the newborn at a referral health facility in Blantyre, Malawi, the Queen Elizabeth Central Hospital (QECH).

## Methods

### Study design and setting

This was a prospective observational study conducted at the Gogo Chatinkha Maternity Unit and the Pediatrics and Child Health Departments at the QECH in Malawi between February 1, 2004 and October 30, 2005. The QECH is a public health facility serving as the referral facility for the southern region of Malawi (population estimated at 5 million), as well as a district hospital for Blantyre (population estimated at 650,000). The Department of Obstetrics and Gynecology at the hospital has often had between 3 and 6 specialist physicians, two to three registrars and 3 to 5 intern medical doctors. There are between two and fours nurse midwives for each shift. There are about six pediatricians at the hospital.

### Study objectives

The objectives of the study were to estimate the incidence of SB and ENND and assess the gestational age, birth weight and sex distribution of the perinatal deaths observed. During the 12 months study period a total of 10,700 deliveries were conducted at the study center.

Consecutive women presenting for labor and delivery care were enrolled. Data were collected on SB and in hospital ENNDs using a standardized and pre-tested data capture sheet. Maternal and fetal variables of interest including GA, BW, sex of fetus or baby, vital status (dead or alive) were collected by a trained research midwife. Information on live births, but who might have died at home after discharge from the hospital, was unknown. No effort was made to follow-up women in the community who did not present for a postnatal visit. Data were analyzed using SPSS statistical software.

Results are presented as frequencies, proportions and graphs where appropriate. The study protocol was reviewed and approved by the University of Malawi's, College of Medicine Research and Ethics Committee (COMREC). All women who participated in the study gave informed consent.

## Results

### Socio-demographic characteristics

Maternal age ranged between 15 and 45 years with a mean of 23 years (SD = 5 years). The majority (89.8%) of the mothers was married and 82% were house wives. Just over half (52%) reported having attended elementary school and 31% had reached secondary school. About one in ten (9%) of mothers did not attend any school and 1.4% had attended higher education. Some 38% of the perinatal deaths had occurred to first pregnancies. The majority, i.e.., 80% (N = 677) of mothers attended at lest two ante-natal (ANC) visits. 15.5% (N = 131) did not attend ANC.

### Prevalece of perinatal mortality

During the 21 months study period (February 1, 2004 to October 30, 2005) there were 10,700 deliveries of whom 845 (7.9%) resulted in perinatal deaths; 4% (N = 363) of all the deliveries were SBs. Of all the perinatal deaths however, these were distributed as 363 (43%) stillbirths and 56% (n = 473) neonatal deaths. A total of 171 (47.1%) of the SBs death occurred before admission to labor ward while 52.9% (192) occurred during labor and delivery process. Data on period of the fetal loss were missing in 9 cases. Most of the women (62.7%) with a perinatal death had at least one previous episode of perinatal death. A total of 85.6% (n = 723) of the deaths were singleton, 13.3% (n = 112) were either or both of twins and the remaining five deaths were triplet deliveries.

### Antenatal laboratory findings

ABO blood group and Rhesus factor (Rh) status were determined in only 42 mothers (5.0%). In 138 (16.4%) mothers (16.4%), VDRL (venereal diseases reference laboratory) for syphilis was done of whom 14 (10.6%) were sero-reactive.

### Gestational ages of Perinatal Deaths

**Figure 1:**
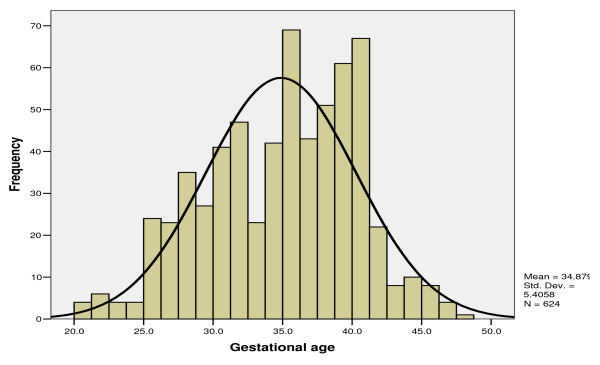
**Distribution of gestational ages in weeks among perinatal deaths at QECH 2004-5**.

Gestational ages (GA) could be calculated on 72.5% (n = 613) of the perinatal deaths based on reported last day of menstrual period and could not be estimated in 27.5% (n = 232) of cases. This was mainly because of unknown or irregular menstrual cycle. Mean GA for perinatal deaths was 34.7 wks (SD = 5.2 wks). The shortest duration of pregnancy recorded was 20 completed weeks; the longest 45.3 weeks. Most (58.9%) of the perinatal deaths were preterm deliveries. Table 1 shows the distribution of the perinatal deaths by gestational age categorized as preterm, term and post term deliveries. The distribution of perinatal deaths by gestational age in weeks is shown in Figure 1.

**Table 1:** Perinatal death by Antepartum, Intrapartum and Neonatal periods in Blantyre, Malawi, 2004-5.

**Period of death of fetus or neonate**	**Frequency (%)**
Antepartum	171 (20.4
Intrapartum	192 (23.0)
Neonatal	473 (56.6)

Total	836 (100.0)

### Birth weight (BW) and perinatal deaths

**Figure 2:**
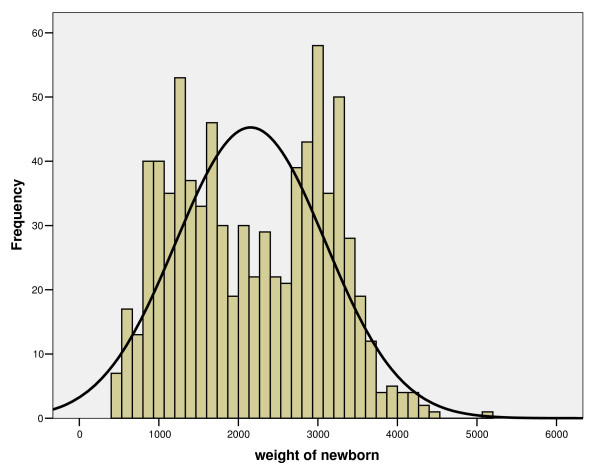
**Birth weight (in grammes) distribution of perinatal deaths**.

Mean birth weight of the perinatal deaths was 2,155 grams (standard deviation = 938 g). Most 56.6% (n = 452) of perinatal deaths weighed between 500 and 2,499 g, i.e. low birth weight; 41.7% (N = 333), between 200 g and 3,999 g. The lowest BW recorded was 500 g and the heaviest newborn weighed 5,150 g. Data on BW were missing in 5.4% (n = 46) of the PD. Figure 2 shows the BW distribution of perinatal deaths.

A total of 468 (55.4%) of perinatal deaths were males, while females accounted for 41.4% (350). The male to female ratio at birth for the perinatal deaths was 1.3:1. A total of 723 (85.6%) of the deaths were singleton, 112 (13.3%) were either or both of twins and the remaining five deaths were triplet deliveries.

## Discussion

Still births and early neonatal deaths are an important and a fairly common outcome among pregnant and postnatal women at the QECH. We found a SB rate of 34/1,000 births which was lower than the 45 deaths per 1,000 births reported from a rural community in Mangochi, southern Malawi [11]. The higher perinatal mortality from Mangochi may be explained by the lack of trained human resources for health (HRH) and inadequate facilities for prenatal, intrapartum and postnatal care [12]. In addition, the Mangochi study was conducted before 2000 and our study was carried out several years later. The passage of time may have been associated with an improvement in maternal and neonatal health overall across the country. Were a more recent study conducted in Mangochi, the results would have provided a better reference to our study.

The prevalence of perinatal deaths that we obtained is also lower than the 124/1000 live births obtained by Kidanto et al [13] at Muhimbili National Hospital, Dar es Salaam (Tanzania) for 1999-2003. In Kenya, Weiner et al reported a perintal mortality rate of 118 per 1000 births in 2003 [5]. Kuti et al, reported a perinatal mortality rate in a Nigerian teaching hospital of 77.03 per 1,000 total births [6]. More than two decades ago, Abudu and Akinkugbe [14] had reported a perinatal mortality rate at 42.5/1000 total births in Lagos, Nigeria. In South Africa however, a much lower perinatal mortality rate of 59 per 1000 births has been reported [15], but still much higher that what is reported from high income countries [16].

The perinatal deaths rate that we found may not be representative of hospitals in Malawi. This is because the QECH is a third level health facility which receives high risk referrals. Pregnant women who present to QECH come from the surrounding neighborhoods of the district of Blantyre, health centers and some patients come from the surrounding districts. These are women who are identified either as high risk or already have intrapartal complications that required advanced obstetric care.

The availability of laboratory services to maternity care women can be assessed by examining the proportion of women who accessed VDRL and Rhesus factor assessment among those who experienced a perinatal outcome. The RH factor and VDRL tests should be routinely done, but not always offered to pregnant women in Malawi due to non-availability of laboratory reagents and human resources. Only 5% and 16.4% women had Rh factor and VDRL tests done. This notwithstanding the fact that national maternity guidelines required all pregnant women to have a VDRL test done. Although we did not assess the contribution of syphilis to perinatal death, it is likely to be a leading factor in Malawi. Among the cases who had syphilis assessed with VDRL, prevalence was 10.6%. This finding is similar to the estimate by Mwapasa et al, at 8% who used multiple reagents, other than a single test as was the case in our study [17]. However, the fact that not all women received laboratory investigations for syphilis, rhesus factors and other tests means that the prevalence estimates for positive test results must be interpreted with caution.

Our study is likely to have underestimated the actual prevalence of prenatal mortality among the cohort that was observed. This is because we did not follow the women into the community. Some of the women may have experienced neonatal deaths after being discharged from hospital. A complete picture could have been obtained if an aggressive follow-up schedule were implemented.

Our findings however are of public health significance, because unlike estimates obtained within intervention studies, we studied the prevalence and the associated features within routine care. These findings are therefore of importance in the design, implementation and evaluation of maternity care improvements at the QECH teaching hospital. However, using routine care in a resource-limited setting as the tool to assess the prevalence and associated factors of perinatal deaths also meant that many other investigations that could explain the deaths were missed. Lack of human resources [18-20], laboratory and pharmaceutical supplies and poor adherence to clinical guidelines are likely to be contributors to the high perinatal mortality in Malawi [21].

## Conclusion

We found the prevalence of perinatal deaths among women attending maternity care at a large teaching hospital in Blantyre, Malawi as 9.8%. Many of the deaths were associated with low birth weight and prematurity. This study showed a high rate of pregnancy wastage during pregnancy, labor and delivery process and after birth. We recommend further studies in this setting to assess the effect of intervention aimed to reduced pregnancy wastage in Malawi.

## Abbreviations

BW: Birth weight; COMREC: College of Medicine Research and Ethics Committee; EHND: early hospital neonatal death; ENND: early neonatal death; GA: gestational age; HRH: human resources for health; PD: perinatal death; PMR: perinatal mortality rate; QECH: Queen Elizabeth Central Hospital; SB: stillbirth; SPSS: Statistical Package for the Social Sciences; VDRL: venereal diseases reference laboratory.

## Competing interests

The authors declare that they have no competing interests.

## Authors' contributions

AMM designed the study, collected data, conducted the analysis and participated in the drafting of the manuscript. ASM conducted some analysis, participated in the interpretation of the results and participated in the drafting of the manuscript.
